# A Study of the Context Upon Community Re-entry for Young Adults With a History of Sexual Offending Sentenced to Shorter Incarcerations

**DOI:** 10.1177/10790632261417991

**Published:** 2026-01-14

**Authors:** Julien Frechette, Patrick Lussier, Isabelle F. Dufour

**Affiliations:** 1 School of Social Work and Criminology, 4440 Université Laval, Quebec, Canada; 2 515168 Centre International de Criminologie Comparée, Montreal, Canada; 3 5599 Institut National de Psychiatrie Légale Philippe-Pinel, Montreal, Canada; 4 Programme de psychoéducation, Université Laval, Quebec, Canada; 5 542830 Institut Universitaire Jeunes en Difficulté, Montreal, Canada

**Keywords:** community correction, community reintegration, individual with sexual offending history, prison, recidivism

## Abstract

Research regarding community re-entry of individuals with sexual offending histories (SOH) has focused on preventing sexual re-offending through surveillance and support interventions. However, most individuals with SOH, serving shorter incarceration sentences, often lack surveillance and support upon community re-entry. This is especially concerning for young adults with SOH as they often are subjected to a negative social reaction in addition to having psychosocial needs and a high level of risk and criminogenic needs, which might result in not receiving tailored interventions, and therefore re-entering the community in a precarious context. Using prospective longitudinal data from a cohort of 1,054 young adults sentenced to shorter incarceration terms in Quebec, Cox regressions were conducted to determine whether there were contextual factors conducive to re-incarceration specific to individuals with SOH (*n* = 69). Findings indicate that there were none specific to individuals with SOH, and rather that persons with and without SOH are similar in many aspects, notably the high re-incarceration rate. These observations raise questions about the context surrounding community re-entry of young adults, regardless of criminal histories. Implications are discussed in the light of the low-risk assumption among individuals sentenced to shorter incarceration terms and the scope of community risk management.

## Introduction

Since the late 1990s, criminal justice systems in the United States (e.g., English, 1998), Canada (e.g., Lussier et al., 2014), and the United Kingdom (e.g., McAlinden, 2006), concerned with the risk of sexual re-offending posed by individuals with sexual offending histories (SOH), have increasingly accepted the need to constrain the liberties of such individuals as the necessary cost of ensuring public safety by introducing targeted community interventions. The American experience with these interventions has been mainly criticized for the initiation of public registration and notification, residence restriction, and GPS monitoring (Lobanov-Rostovsy, 2015). Researchers have raised ethical, legal, and social concerns about such interventions, particularly in relation to unintended negative consequences and secondary punishment for individuals with SOH following community re-entry (Durling, 2006; Levenson et al., 2016; Socia, 2021). While most countries have not enacted the same criminal justice policies as the United States, relying on more modest interventions (e.g., Bartels et al., 2021; McCartan & Richards, 2021), studies of the American experience are useful in highlighting the importance of understanding the context surrounding community re-entry and its influence on re-offending for persons with SOH (Burchfield & Mingus, 2008; Cann & Isom Scott, 2020; Duwe & Donnay, 2010). Building on these observations, particularly as there is little research available in other countries, the current study examines the context upon community re-entry for persons with SOH in Canada. The Canadian experience appears different from that of the United States given its emphasis on (a) treatment and rehabilitation, (b) individualization in sentencing, and (c) community corrections and risk management (Lussier & McCuish, 2024; Meyer & O’Malley, 2005; Tonry, 2013). Therefore, prospective cohort data were used to examine whether the legal, interventional, and psychosocial context in Canada surrounding the community re-entry of individuals with SOH is conducive to re-offending and, if so, whether this context is specific to them as opposed to persons without such histories.

### The Social Reaction to Persons With Sexual Offending Histories

Canadians, like their American counterparts, tend to have negative attitudes about individuals with SOH and positive views about restrictions on community re-entry, such as requiring public registration and notification as well as residence restrictions to prevent re-offending (Corăbian et al., 2024; Jung et al., 2018; Knack et al., 2021). These observations merit more attention from a research perspective given a Canadian study found that many individuals with SOH feel that having personal information made public increases the risk of re-offending, particularly given the collateral effects of being “outed” (e.g., fear and anger; Murphy & Fedoroff, 2013). Several studies conducted outside Canada have also suggested that a negative social reaction to persons with SOH could have collateral consequences, making community re-entry more complex (for a review of such work, see Tuschick et al., 2024). Hence, the criminal justice system, which is responsible for both public safety and the social reintegration of persons with SOH, is faced with a conundrum in terms of community re-entry and management of this group, that is finding a way to reconcile demands that the concerns of citizens be taken seriously, particularly given the nature of the risk involved (i.e., sexual re-offending), while also taking into consideration the expertise of researchers on risk, desistance, and social reintegration to promote successful community re-entry for individuals with SOH.

### Community Correction Interventions: The Pillars of Canadian Risk Management

Taking into account Canada’s long tradition of scientific research regarding risk and treatment (Marshall & Laws, 2003), the criminal justice system reaffirmed the ideal of rehabilitation rather than relying on incapacitation and informal social control in dealing with the community re-entry of individuals with SOH (Petrunik et al., 2008). Research on effective treatment programs (e.g., Dowden & Andrews, 2000; Gendreau & Ross, 1979) as well as on the potential harmful effects of long incarcerations (e.g., Heskin et al., 1973) have led the Canadian criminal justice system to favor community correction, regardless of an individual’s criminal history.

In Canada, as well as several other Western countries, community re-entry and risk management of those with and without SOH relies on formal surveillance and support interventions to prevent re-offending (e.g., Correctional Service Canada, 2019; Duan et al., 2024; Kemshall & McCartan, 2014). Community correction leans on practitioners in criminal justice, working within a system of sentencing and conditional release, such as probation and parole, to simultaneously monitor the whereabouts and adherence to legal conditions of persons who have been returned to the community while also offering rehabilitation services. Their interventions, guided by risk and criminogenic needs principles, are tailored to each individual’s probability of re-offending as well as factors contributing to criminal behaviors (Andrews et al., 1990). The level of risk and criminogenic needs is determined through a risk assessment conducted by a trained criminal justice practitioner, who relies on a careful examination of correctional files and in-depth clinical interviews (Bonta et al., 2021; Guay, 2016). Such risk assessments and the ensuing surveillance and support interventions have been shown, notably in Canada, to prevent re-offending for persons with or without SOH (Arbour & Marchand, 2022; Bonta & Andrews, 2007; Hanson et al., 2009; Motiuk & Keown, 2021). Research on community re-entry has, however, focused on risk assessments designed to estimate the risk of re-offending, especially the risk of sexual re-offending of individuals with SOH, while the broader context in which community re-entry occurs has gained less attention.

### The Community Re-entry Context After Shorter Incarcerations (Less Than Two-Years)

Individuals sentenced to shorter^1^ incarcerations, known in Canada as provincial sentences and lasting less than two years, may not receive surveillance and support interventions upon community re-entry. This legal context upon community re-entry, which also occur in other countries (e.g., Lievesley et al., 2018), is concerning because the majority of incarceration sentences for individuals with or without SOH, in both Canada and other Western countries, fall within the less than two-year range (e.g., Hanson et al., 2012; Ministry of Justice, 2014; Statistics Canada, 2024). Of importance, young adults with SOH often receive such shorter sentences, despite their higher risk of re-offending and numerous criminogenic and psychosocial needs (e.g., Craig, 2011; Fougere et al., 2013; Hanson & Bussiere, 1998; Wilson & Yates, 2009). In line with the extensive scientific literature on the risk of persons with SOH, surveillance and support interventions provided upon release into the community are perhaps not suited to their level of risk and criminogenic needs, which may increase their probabilities of re-offending (Andrews et al., 2004). Yet, this discrepancy between individual intervention needs and those delivered has been observed in studies suggesting the use of overly intensive sexual re-offending prevention interventions given the level of risk and the nature of criminogenic needs of some individuals with SOH (Lussier & Frechette, 2022; Mailloux et al., 2003).

Complementarily, other researchers suggest that individuals with SOH also return to the community in a psychosocial context that is conducive to re-offending, as there are multiple barriers to social reintegration (e.g., Ward et al., 2007; Wilson & Yates, 2009). Reviews of the empirical literature has shown that individuals with SOH encounter three main barriers upon re-entry: negative social reactions (e.g., stigmatization), interfering and inadequate responses by the criminal justice system (e.g., community correction interventions), and difficulties in meeting psychosocial needs such as housing, employment, mental health and other social or personal issues (Grossi, 2017; Tuschick et al., 2024). In addition, American studies suggest that individuals with SOH encounter more barriers than individuals without such histories (e.g., Allan et al., 2023; Rydberg, 2018). However, while the compounded effect of these barriers and their influence on re-offending is relatively well documented in the United States (e.g., Cann & Isom Scott, 2020; Levenson & Cotter, 2005; Mercado et al., 2008), this area remains relatively unresearched in Canada. The limited literature available notes the presence of certain barriers (e.g., Dubois & Ouellet, 2020; Murphy & Fedoroff, 2013; Ricciardelli et al., 2023), but their simultaneous influence on re-offending among individuals with SOH, particularly compared to persons without SOH, is unknown.

### Research Aim

Prior research suggests that beyond individual factors, the legal, interventional, and psychosocial context surrounding community re-entry for young adults with SOH sentenced to shorter incarceration terms may increase the likelihood of re-offending, more than those without such histories. Indeed, individuals with SOH seem to experience more negative social reactions upon community re-entry than other persons, even in Canada, where conversely to the American experience, there are no public registration and notification or residence restriction. However, it remains unclear if and how context is conducive to re-offending and whether some effects of context are specific to young adults with SOH. Consequently, the present study is aimed at determining whether, for young adults sentenced to shorter incarceration terms, there are contextual factors conducive to re-offending specific to individuals with SOH compared to those without SOH. The research examined the effect of the context through (1) community correction surveillance and support as reflected by probation and parole status, (2) community intervention needs with regard to intensity and programming, and (3) psychosocial needs arising from housing, employment, mental health, and social difficulties. Using a prospective cohort study design, data was collected on criminal history, risk assessment, and community correction for a representative sample of young Canadian adults sentenced to a shorter incarceration term. The data was subjected to a series of survival analyses to allow comparison of persons with SOH and persons without SOH in terms of time to re-incarceration and associated contextual factors.

## Method

### Sample

The sample was a cohort of 1,430 adult men aged 18 to 34 years old who had been admitted consecutively to a provincially run institution in the Province of Quebec between April 1, 2009, and March 31, 2011, to serve a sentence of six months to two years less a day. Less than one in ten had SOH. A representative sample of the correctional population in that age-group sentenced to prison in the Province of Quebec, the participants were part of a larger research project on offending by young adults and desistance trajectories. Individuals serving a federal sentence in a provincial institution (*n* = 165) were excluded from the study sample, as were individuals whose index incarceration conviction did not involve the Quebec correctional services (*n* = 22), individuals who were still incarcerated at the end of the follow-up period (*n* = 2), and individuals who lacked a completed risk assessment for their index incarceration as their admission date was before or after the period of data collection (*n* = 184). Finally, as the study sample included mainly individuals whose SOH involved physical contact, three individuals whose convictions were only for non-physical contact were excluded from the study to avoid possible bias (see Faust et al., 2015). The final study sample therefore included 1,054 individuals, 6.5% of whom had SOH (*n* = 69), a percentage that has been found to be representative and stable across time in provincial institutions in the Province of Quebec (Ministère de la Sécurité publique du Québec, 2024).

### Procedure

In line with the prospective cohort design, individuals sampled upon admission to prison were followed up both in custody and in the community by correctional services, resulting in the compilation of data up to December 31, 2015, at which point a single extraction of correctional files was performed from the computer system of the Quebec correctional services (DACOR). The files contained an exhaustive history of each individual’s contact with the Quebec correctional services until that point, including information regarding incarceration and community sentences. Appended to the files was a risk assessment carried out shortly before the individual became eligible for parole, which included recommendations for parole boards and guidance for parole agents in the community on how to provide adequate surveillance and support intervention planning before a potential re-entry into the community. The risk assessment instrument used was the Level of Service and Case Management Inventory (LS/CMI; Andrews et al., 2004), which has both an actuarial and a community management component. The psychometric properties of the actuarial component of the LS/CMI, capturing the level of risk and criminogenic needs, has been widely documented, particularly in Canada (Giguère & Lussier, 2016; Olver et al., 2014). Risk assessments were not undertaken for research purposes but were completed by criminal justice practitioners as part of their professional duties. The data was anonymized by the Ministry of Public safety, making it impossible to identify persons in the sample from the extracted information. This research project was granted ethical and operational approval from the different institutions involved.

### Study Variables

#### Contextual Factors

This study examines the key role of contextual factors in shaping re-offending upon community re-entry, focusing on three components: (1) community correction surveillance and support, (2) community intervention needs, and (3) psychosocial needs.

Community correction surveillance and support referred to the probation and parole status of the individual being released into the community. The focus was on the legal mandates of probation and parole related to the index incarceration. Almost one in two persons had been released on probation (47.6%; *n* = 502), 10.1% had been released on parole (*n* = 106), and 42.3% had been released without probation or parole (*n* = 446). For the analyses, time-dependent variables were used to account for the duration of the legal mandates related to probation and parole. The average duration of the legal mandate for probation was 1.83 years (*SD* = 0.72) and 0.96 years (*SD* = 0.35) for parole, with individuals released without probation or parole serving as the reference group, as they were not under a legal mandate upon community re-entry for the index incarceration. Note that probation includes individuals whose surveillance and support was mandated as well as those where only surveillance was required as, due to the nature of the data, it was impossible to separate these aspects.

Community intervention needs reflected the recommendations of criminal justice practitioners provided when parole is being considered. In contrast to the level of risk and criminogenic needs resulting from the actuarial component of the LS/CMI, which is aimed at preventing re-offenses, recommendations by criminal justice practitioners may also incorporate considerations of risk management and social reintegration beyond criminal behaviors and related factors (e.g., available resources in remote communities, unexpected urgent daily life situations that require immediate intervention). Two variables taken from the “Program and placement decision” section of the LS/CMI were examined: high intensity intervention recommended (0 = No; 1 = Yes) and no program recommended (0 = recommended; 1 = not recommended). The intensity of intervention was determined based on the recommended frequency of meetings with the individual being released. The default frequency was once a month, with individuals for whom more meetings were recommended assigned to the group of high intensity intervention, which included 66.5% of the sample (*n* = 701). The programming variable included not only therapeutic programs but also programs related, for instance, to finding recreational activities or jobs, recovering from substance abuse, and obtaining social assistance. A total of 44.3% had not been recommended for program participation (*n* = 467). However, many of the recommendations made by criminal justice practitioners might have not materialized from a formal intervention standpoint given almost four out of 10 individuals who had been recommended for high intensity and/or therapeutic programs re-entered the community without probation or parole (calculation not shown).

Psychosocial needs, which complement community intervention needs, referred to an individual’s specific difficulties in meeting the normative demands of daily life in society observed in anticipation of community re-entry by criminal justice practitioners conducting the risk assessment. The psychosocial needs were also retrieved from the LS/CMI, largely from the sections “Other client issues” as well as “Responsivity considerations”, and therefore corresponded to items that do not contribute directly to the actuarial score for the level of risk and criminogenic needs. The four variables examined were: housing difficulties, employment difficulties, mental health issues, and low social abilities (rated as 0 = No; 1 = Yes). Employment difficulties^2^ and low social abilities were not the result of a transformation. However, housing difficulties was a combination of the dichotomous items “Homeless/transient” and “Accommodation problems”, while mental health issues was a combination of the dichotomous items “Diagnosis of serious mental disorder”, “Mental disorder”, and “Antisocial personality/psychopathy”. If any of the items was present, the psychosocial need was coded as present (1 = Yes). The variables used thus result from the combination of selected items based on theoretical considerations and practical relevance rather than statistical procedures. Descriptive analyses revealed that the most recurrent psychosocial need upon community re-entry was related to employment (48.6%; *n* = 512), followed by housing difficulties (17.6%; *n* = 186), low social abilities (14.2%; *n* = 150), and mental health issues (10.2%; *n* = 108).

Community intervention needs and psychosocial needs, although dynamic in nature, were measured at only one time point as periodic reassessment of such factors was not common practice, and therefore, not available. This limitation is somewhat offset by the relative (in)stability of these difficulties and recommendations (e.g., Gustavson et al., 2007; Herbert et al., 2015; Van der Geest et al., 2011). Indeed, they generally refer to needs that are hardly resolved within a few months (or even years), often requiring sustained intervention, well beyond the realm of the criminal justice system.

#### Individual Factors

While the context surrounding community re-entry is a central component of the present study, individual factors also had to be controlled for, given their association with re-offending. The level of risk and criminogenic needs obtained via the actuarial score of the LS/CMI was therefore also included in the analyses. Scores range from 0 to 43, with 0 indicating very low risk and needs and 43 very high risk and needs. Mean actuarial score was 28.0 (*SD* = 7.3), which corresponds to a high level of risk and criminogenic needs according to the guidelines of the LS/CMI. For the analyses, the level of risk and criminogenic needs was treated as a binary variable: low-moderate (0) or high (1). For the overall sample, 84.8% had a high level of risk and criminogenic needs upon community re-entry (*n* = 894). A variable capturing the number of prior incarcerations was also examined, using information retrieved from the correctional files. At the time of community re-entry for the index incarceration, individuals in the sample had on average been incarcerated 2.2 times since turning 18 (*SD* = 2.2). Given the asymmetric distribution of the number of prior incarcerations (median = 1.0; range = 0 – 24), the variable was transformed onto a natural logarithmic scale whose geometric mean was 0.3 (*SD* = 0.3). As it was expected that the number of prior incarcerations would be strongly correlated to the age of the individual, a variable not examined (and not available), a quadratic effect was used. Adding this effect was important, since the age of the older individuals in the sample coincided with the age that has been found to be associated with the beginning of a decline in criminal behavior in adulthood (e.g., Farrington, 2003), which could in turn influence the shape of the effect of the number of prior incarcerations on re-offenses.

#### Re-incarceration

In order to examine the effect of contextual factors on re-offending, follow-up data on re-incarcerations between April 1, 2009 and December 31, 2015 were retrieved from the correctional files. During a mean follow-up period of 4.7 years (*SD* = 0.6, range = 1.2 – 5.8), 775 persons were re-incarcerated (73.5%). Among these re-incarcerations, 10.0% were due to new convictions for a violent crime (*n* = 77), while 21.3% resulted from convictions for property crimes (*n* = 165), 21.9% for other crimes (e.g., drug, traffic, municipal by-law; *n* = 170) and 46.8% for back-door sentencing measures (i.e., correctional decisions rather than court; *n* = 363). Hence, re-incarceration includes both those as a result of a new offense (35.6%; *n* = 375) and a breach of conditions (38.0%; *n* = 400). About half of re-incarcerations for a breach of conditions (51.2%; *n* = 205) were related to probation and parole legal mandates for the index incarceration, while the other half stemmed from a breach that was not associated with the index incarceration but with a subsequent community sentence. Analyses were conducted separately for the outcome of any re-incarcerations, re-incarcerations resulting from a new offense, and re-incarcerations resulting from a breach of conditions.

### Analytical Strategy

#### Survival Analyses

Non-parametric survival analyses (Kaplan & Meier, 1958) were conducted to determine whether there were statistically significant differences in the time-to-event of re-incarceration outcomes between groups. Survival analyses incorporate this temporal component by taking into consideration the varying time-at-risk periods for individuals, which referred in the present study to the period between release from incarceration and re-incarceration. Given that some individuals were not re-incarcerated during the follow-up period but might be re-incarcerated later, this method was designed to handle the right-censored data common to re-offense studies (Lussier & McCuish, 2016). The results of survival analyses were presented using Kaplan Meier Curves (Kaplan & Meier, 1958). To compare curves corresponding to the survival function of individuals with and without SOH, Log-Rank (Mantel-Cox) tests, which assume equal weight throughout the time points, were performed. Also, and most importantly, Cox regressions were conducted to examine whether there are contextual factors conducive to re-incarceration outcomes specific to persons with SOH as opposed to those without such histories while controlling for the effect of individual factors (Cox, 1972).

##### Competing Risk

Cox regressions were carried out using a two-step approach to account for the competing risks inherent to re-incarcerations, that is that re-incarceration for a new offense prevents re-incarceration for a breach of conditions and vice-versa (Rhodes et al., 2025). The first regression for any re-incarcerations combined re-incarceration for a new offense and re-incarceration for a breach of conditions, treating non-re-incarcerated persons as censored. The second regression estimated re-incarcerations for a new offense, considering individuals re-incarcerated for a breach of conditions and persons not re-incarcerated as censored. Using the same approach, a final regression dealing with re-incarcerations for a breach of conditions was conducted where persons re-incarcerated for a new offense and persons not re-incarcerated were censored.

##### Modeling

In order to distinguish whether certain contextual factors contribute to re-incarceration specifically for individuals with SOH, study covariates were introduced into the regression analyses using a nested model approach. The modeling strategy was designed to sequentially estimate the main effect of the presence of SOH under the assumption of homogeneous effects of individual and contextual factors across persons with and without such histories (Models 1–2) and then allow these effects to vary between the two groups (Model 3). Therefore, Model 1 included individual factors – level of risk and criminogenic needs as well as the number of prior incarcerations and its quadratic effect – in addition to the presence of SOH.

Model 2 added contextual factors – community correction, community intervention needs, and psychosocial needs covariates. However, to account for unequal exposure to community correction legal mandates, time-dependent probation and parole covariates were specified using the segmented time-dependent covariate dialog box of the software Statistical Package for the Social Sciences (IBM Corporation, 2023). This approach, referred to as the programming statements method, used a logical expression – i.e., T_ ≤ number of days the [insert legal mandate] was active – which took the value 1 during exposure to the legal mandate and 0 thereafter (Allison, 2014). Because the study focused on the effect of probation and parole related to the index incarceration, a single logical expression was defined for each legal mandate, enabling comparisons with the reference group of persons released without probation or parole (value of 0 throughout). This approach was appropriate as values of probation and parole covariates did not vary continuously over time but were defined by a single event for which time boundaries were known: starting at community re-entry, and ending instantaneously when the legal mandate became inactive. Hence, these covariates, which remained constant within their respective time intervals, followed a step-function specification. This ensured that the effect of each legal status upon community re-entry on re-incarceration outcomes was estimated only while it remained active for individuals released on probation and parole (Allison, 2014; Therneau et al., 2017). Indeed, given that surveillance within community correction is intrinsically tied to the duration of the probation and parole legal mandates, meaning that it can only exert an effect while these mandates are active, the step function was preferred over time-lag or weight functions, which assume persisting, increasing or even fading effects that seemed more speculative (see Fisher & Lin, 1999). This method also allowed valid comparisons between individuals released from incarceration for the index incarceration on parole, on probation, or without parole or probation, as the outcome was not restricted to those for whom community interventions for the index incarceration had been legally mandated. Community intervention needs and psychosocial needs introduced in Model 2 were instead time-independent covariates (value does not change over time), thus requiring no specific manipulation or function in the Cox regressions. Finally, Model 3 introduced interaction terms between the presence of SOH and individual and contextual factors. No multicollinearity problems were identified within the study variables.

Consistent with the study objective, model-fit comparisons were conducted to examine whether allowing the effects of contextual factors to vary across persons with and without SOH yielded a more optimal solution compared to assuming homogeneous effects for the overall sample. The Bayesian Information Criterion (BIC; Schwarz, 1978) was compared across the models, with special attention given to the comparison between Model 2 and Model 3. The BIC is a model-fit statistic that accounts for model parsimony by penalizing more complex models. Lower BIC values indicate better trade-off between fit and parsimony. This step ultimately guided interpretation by indicating which model was the most generalizable and efficient. In fact, the BIC value difference was interpreted in light of Raftery’s (1995) guidelines for model selection in social research where differences between 0-2, 2-6, 6-10, and more than 10 were respectively weak, positive, strong, and very strong. Hence, a lower BIC value for Model 3 compared to Model 2 would suggest that some effects of contextual (and individual) factors differ between persons with SOH and those without such histories.

The authors take full responsibility for the integrity of the data as well as the accuracy of the analyses and have made every effort to avoid inflating statistically significant results while reporting as much information as possible about the manipulations of data to ensure transparency and reproducibility.

## Findings

Prior to survival analyses, bivariate analyses were performed to examine the distribution of study variables within and between the groups under scrutiny (Table 1). The only significant association observed was with probation for the index incarceration: persons with SOH (60.9%) were proportionally more likely than persons without such offending histories (46.7%) to be sentenced to probation for their index incarceration (*X*^*2*^ = 5.19, *p* = 0.023, *d* = 0.14). The length of the legal mandate in the community among individuals on probation for the index incarceration was longer for individuals with SOH than those without (*t* = - 2.32, *p* = 0.01, *d* = 0.37). The strength of both these differences, however, was relatively weak (Cohen, 1988).

**Table 1.** Descriptive Statistics of Study Variables

	Overall *n* = 1,054	Persons w/SOH *n* = 69	Persons w/out SOH *n* = 985	
	*n* (%)/M (SD)	*n* (%)/M (SD)	*n* (%)/M (SD)	d
Individual factors				
Lvl of risk-crim. needs (high)	894 (84.8)	61 (88.4)	833 (84.6)	.05
No. prior incarcerations^a^	2.2 (2.2)	2.5 (2.2)	2.18 (2.2)	.18
No. prior incarcerations sq.^b^	1.3 (1.4)	1.3 (1.4)	1.31 (1.4)	.04
Contextual factors				
Probation (yes)	502 (47.6)	42 (60.9)	460 (46.7)	.14*
Duration (in years)^c^	1.83 (0.72)	2.08 (0.72)	1.81 (0.71)	.37**
Parole (yes)	106 (10.1)	5 (7.2)	101 (10.3)	.05
Duration (in years)^c^	0.96 (0.35)	0.94 (0.46)	0.96 (0.34)	.04
No probation or parole	446 (42.3)	22 (31.9)	424 (43.0)	.11
High intensity rec. (yes)	701 (66.5)	45 (65.2)	656 (66.6)	.01
No program rec. (yes)	467 (44.3)	26 (37.7)	441 (44.8)	.07
Housing difficulties (yes)	186 (17.6)	14 (20.3)	172 (17.5)	.04
Employment difficulties (yes)	512 (48.6)	31 (44.9)	481 (48.8)	.04
Mental health issues (yes)	108 (10.2)	11 (15.9)	97 (9.8)	.09
Low social abilities (yes)	150 (14.2)	11 (15.9)	139 (14.1)	.03
Sexual offense history	69 (6.5)	-	-	-
Re-incarceration				
Any re-incarceration (yes)	775 (73.5)	48 (69.6)	727 (73.8)	.05
Re-incarceration new offense (yes)	375 (35.6)	28 (40.6)	347 (35.2)	.05
Re-incarceration breach (yes)	400 (38.0)	20 (29.0)	380 (38.6)	.09

Notes. *p ≤ 0.05; **p ≤ 0.01.

^a^As the distribution of the number of prior incarceration is positively skewed, a log transformation was used; reverse log coefficients are reported in the table.

^b^The log transformed variable of the number of prior incarcerations was centered, then squared to avoid multicollinearity problems.

^c^Descriptives regarding duration were reported for persons released for the given type of surveillance and support.

### Survival Curves

**Figure 1.**
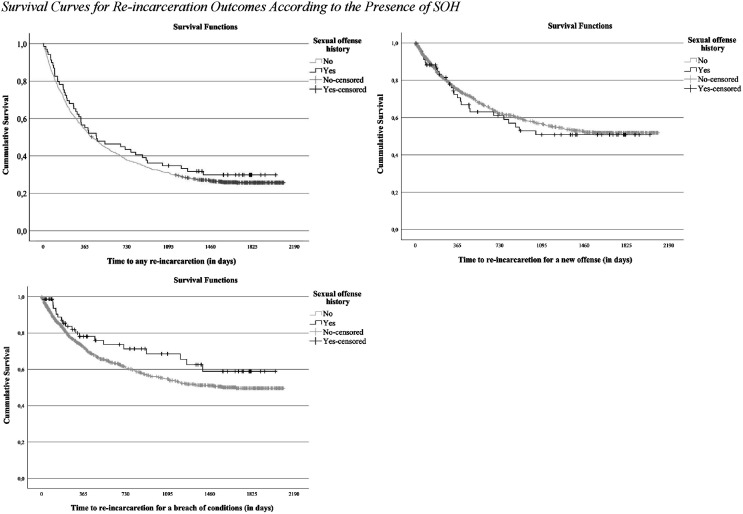
Survival curves for re-incarceration outcomes according to the presence of SOH

Kaplan-Meier survival analyses found no statistically significant differences between persons with SOH and persons without SOH in terms of the expected and observed survival time for any re-incarceration, re-incarceration for a new offense, and re-incarceration for a breach of conditions (Figure 1). Concerning any re-incarcerations, persons with SOH had a mean survival time of 2.41 years (*SE* = 0.27) as opposed to 2.24 (*SE* = 0.07) for persons without SOH (Mantel-Cox = 0.693, *df* = 1, *p* = 0.408). Slightly over half of the overall sample had been re-incarcerated within the first year, 62.5% (*n* = 486) of all individuals re-incarcerated during the follow-up period (calculation not shown). The slope then becomes less and less steep over time for both groups, indicating that any re-incarcerations were becoming less likely. Very similar findings were observed for re-incarcerations for a new offense (Mantel-Cox = 0.152, *df* = 1, *p* = 0.697). Individuals with SOH had a mean survival time (*M* = 3.34, *SE* = 0.31) similar to that of individuals without SOH (*M* = 3.58, *SD* = 0.09). Finally, even though there was no significant difference between individuals with SOH and individuals without SOH with regard to re-incarceration for a breach of conditions, individuals without SOH outpaced individuals with such histories in being re-incarcerated sooner (Mantel-Cox = 2.34, *df* = 1, *p* = 0.126). Precisely, individuals with SOH (*M* = 3.88 years; *SE* = 0.30) were re-incarcerated for a breach of conditions on average almost half a year later than persons without SOH (*M* = 3.45, *SE* = 0.09). If the relatively steep slope during the first follow-up year was similar between the two groups, the gap seemed to widen during the second and third year before slowly becoming closer and then reaching a plateau in year four and five.

### Competing Risk Cox Regression With Time Dependent Covariates

Bivariate analyses suggested that individuals with SOH and individuals without SOH have similar individual and contextual factors profiles upon community re-entry as well as a similar time-to-event across the different re-incarceration outcomes. However, more complex analyses using Cox regressions were conducted in order to determine whether certain contextual factors were specifically conducive to re-incarcerations for persons with SOH. The results are presented in Tables 2–4.

**Table 2.** Regression Table of the Competing Risk Cox Regression Analyses for Any Re-Incarceration

	Model 1 Individual factors			Model 2 Contextual factors			Model 3 Interaction effects		
	HR	95% CI	*p*	HR	95% CI	*p*	HR	95% CI	*p*
Sex. offense history (SOH)	0.83	0.62-1.12	.224	0.84	0.63-1.13	0.253	1.26	0.30-5.24	.747
Lvl of risk-crim. needs	2.44^***^	1.89-3.15	≤.001	1.98^***^	1.49-2.64	≤.001	2.01^***^	1.49-2.70	≤.001
No. prior incarcerations	1.68^***^	1.31-2.15	≤.001	1.67^***^	1.29-2.14	≤.001	1.60^***^	1.23-2.07	≤.001
No. prior incarcerations sq.	1.23	0.67-2.27	.509	1.11	0.60-2.06	.734	1.26	0.66-2.40	.486
Probation^a^				1.12	0.95-1.32	.165	1.15	0.97-1.36	.101
Parole^a^				1.36^*^	1.02-1.81	.038	1.35^*^	1.01-1.81	.046
High intensity rec.				1.17	0.97-1.41	.100	1.16	0.95-1.40	.140
No program rec.				1.04	0.89-1.22	.590	1.09	0.93-1.28	.295
Housing difficulties				1.43^***^	1.19-1.72	≤.001	1.39^***^	1.15-1.68	≤.001
Employment difficulties				1.27^***^	1.10-1.47	≤.001	1.27^**^	1.09-1.48	.002
Mental health issues				1.23	0.98-1.54	.069	1.27^*^	1.00-1.61	.046
Low social abilities				1.08	0.88-1.32	.440	1.08	0.88-1.33	.467
SOH × lvl risk-crim. needs							1.05	0.24-4.71	.946
SOH × no. prior inc.							3.33	0.88-12.67	.077
SOH × no. prior inc. sq.							0.15	0.01-1.94	.145
SOH × probation^a^							0.54	0.26-1.10	.091
SOH × parole^a^							1.36	0.33-5.60	.673
SOH × high intensity rec.							1.04	0.46-2.36	.933
SOH × No program rec.							0.33^**^	0.16-0.69	.003
SOH × housing diff.							2.05	0.90-4.68	.089
SOH × employment diff.							0.88	0.45-1.73	.723
SOH × mental health issues							0.49	0.20-1.17	.109
SOH × low social abilities							0.94	0.39-2.29	.898
Model fit	*X*^*2*^ (4) = 101.53, *p* ≤ .001 BIC = 10,001.12			*X*^*2*^ (12) = 154.91, *p* ≤ .001 BIC = 9,896.79 (−104.33)			*X*^*2*^ (23) = 170.85, *p* ≤ .001 BIC = 9,952.83 (56.04)		
Improvement (*X*^2^)				*X*^*2*^ (8) = 45.88, *p* ≤ .001			*X*^*2*^ (11) = 17.14 *p* = .104		

Notes. *p ≤ .05; **p ≤ .01; ***p ≤ .001.

^a^The reference group corresponded to individuals released without probation or parole.

**Table 3.** Regression Table of the Competing Risk Cox Regression Analyses for Re-Incarceration for a New Offense

	Model 1 Individual factors			Model 2 Contextual factors			Model 3 Interaction effects		
	HR	95% CI	*p*	HR	95% CI	*p*	HR	95% CI	*p*
Sex. offense history (SOH)	1.00	0.68-1.47	.999	0.98	0.66-1.44	.916	2.53	0.32-19.85	.378
Lvl of risk-crim. needs	3.97***	2.51-6.29	≤.001	2.89***	1.76-4.72	≤.001	2.95***	1.78-4.90	≤.001
No. prior incarcerations	2.16***	1.50-3.11	≤.001	1.98***	1.37-2.86	≤.001	1.92***	1.32-2.80	≤.001
No. prior incarcerations sq.	0.98	0.41-2.32	.956	0.89	0.37-2.13	.799	0.90	0.36-2.28	.826
Probation^a^				1.05	0.83-1.31	.685	1.08	0.86-1.37	.506
Parole^a^				0.24**	0.10-0.59	.002	0.25^**^	0.10-0.62	.003
High intensity rec.				1.19	0.91-1.55	.211	1.19	0.90-1.57	.223
No program rec.				1.04	0.83-1.30	.726	1.11	0.88-1.40	.377
Housing difficulties				1.58***	1.22-2.04	≤.001	1.51**	1.16-1.98	.002
Employment difficulties				1.14	0.92-1.40	.222	1.14	0.91-1.41	.249
Mental health issues				1.42*	1.05-1.94	.024	1.43*	1.04-1.98	.030
Low social abilities				1.04	0.78-1.40	.769	1.08	0.80-1.46	.606
SOH × lvl risk-crim. needs							0.74	0.07-7.51	.799
SOH × no. prior inc.							2.78	0.48-16.06	.253
SOH × no. prior inc. sq.							0.47	0.02-12.53	.651
SOH × probation^a^							0.41	0.16-1.04	.060
SOH × parole^a^							0.00	b	.947
SOH × high intensity rec.							0.94	0.29-2.99	.911
SOH × no program rec.							0.27*	0.10-0.74	.011
SOH × housing diff.							2.42	0.82-7.19	.111
SOH × employment diff.							0.93	0.37-2.33	.876
SOH × mental health issues							0.65	0.22-1.95	.445
SOH × low social abilities							0.50	0.13-1.89	.307
Model fit	*X*^*2*^ (4) = 84.78, *p* ≤ .001 BIC = 4,839.11			*X*^*2*^ (12) = 125.86, *p* ≤ .001 BIC = 4,747.63 (−91.48)			*X*^*2*^ (23) = 140.44, *p* ≤ .001 BIC = 4,800.54 (52.91)		
Improvement (*X*^2^)				*X*^*2*^ (8) = 41.27, *p* ≤ .001			*X*^*2*^ (11) = 12.28, *p* = .343		

Notes. *p ≤ .05; **p ≤ .01; ***p ≤ .001

^a^The reference group corresponded to individuals released without probation or parole.

^b^The 95% CI was too large to fit in the table.

**Table 4.** Regression Table of the Competing Risk Cox Regression Analyses for Re-Incarceration for a Breach of Conditions

	Model 1 Individual factors			Model 2 Contextual factors			Model 3 Interaction effects		
	HR	95% CI	*p*	HR	95% CI	*p*	HR	95% CI	*p*
Sex. offense history (SOH)	0.68	0.43-1.06	.089	0.70	0.44-1.10	.120	0.77	0.10-6.08	.808
Lvl of risk-crim. needs	1.80***	1.32-2.6	≤.001	1.57*	1.09-2.26	.015	1.60*	1.10-2.32	.015
No. prior incarcerations	1.33	0.94-1.88	.102	1.41	0.99-2.00	.052	1.35	0.95-1.93	.098
No. prior incarcerations sq.	1.49	0.62-3.56	.370	1.36	0.57-3.29	.488	1.71	0.69-4.20	.245
Probation^a^				1.20	0.95-1.51	.128	1.22	0.96-1.54	.105
Parole^a^				2.50***	1.80-3.45	≤.001	2.45***	1.76-3.42	≤.001
High intensity rec.				1.16	0.90-1.51	.250	1.14	0.87-1.49	.340
No program rec.				1.05	0.85-1.30	.642	1.08	0.87-1.34	.505
Housing difficulties				1.30	0.99-1.68	.052	1.28	0.98-1.67	.071
Employment difficulties				1.42***	1.16-1.74	≤.001	1.42***	1.15-1.75	≤.001
Mental health issues				1.04	0.74-1.45	.828	1.11	0.79-1.56	.561
Low social abilities				1.14	0.86-1.50	.370	1.09	0.82-1.46	.543
SOH × lvl risk-crim. needs							1.19	0.15-9.57	.872
SOH × no. prior inc.							3.47	0.39-30.84	.264
SOH × no. prior inc. sq.							0.01	0.01-1.50	.072
SOH × probation^a^							0.93	0.30-2.93	.908
SOH × parole^a^							2.79	0.51-15.12	.235
SOH × high intensity rec.							0.99	0.28-3.53	.988
SOH × No program rec.							0.47	0.15-1.43	.183
SOH × housing diff.							1.47	0.39-5.50	.566
SOH × employment diff.							0.82	0.28-2.40	.723
SOH × mental health issues							0.21	0.04-1.13	.069
SOH × low social abilities							2.52	0.64-9.97	.188
Model fit	*X*^*2*^ (4) = 28.50, *p* ≤ .001 BIC = 5,181.08			*X*^*2*^ (12) = 81.32, *p* ≤ .001 BIC = 5,151.84 (−29.24)			*X*^*2*^ (23) = 91.84, *p* ≤ .001 BIC = 5,204.93 (53.09)		
Improvement (*X*^2^)				*X*^*2*^ (8) = 47.90, *p* ≤ .001			*X*^*2*^ (11) = 12.83, *p* = .305		

Notes. *p ≤ .05; **p ≤ .01; ***p ≤ .001

^a^The reference group corresponded to individuals released without probation or parole.

#### Any Re-incarceration

The results indicated that Model 1, including individual factors (*X*^*2*^ = 101.53, *df* = 4, *p* ≤ .001), and Model 2, with contextual factors added (*X*^*2*^ = 154.91, *df* = 12, *p* ≤ .001), were significantly associated with hazards of any re-incarceration (see Table 2), although the presence of SOH was not significant. In Model 1, the level of risk and criminogenic needs (*HR* = 2.44, *p* ≤ .001) and the number of prior incarcerations (*HR* = 1.68, *p* ≤ .001) were significantly and positively associated with hazards of any re-incarceration. In Model 2, the insertion of contextual variables surrounding community re-entry significantly improved model fit (*X*^*2*^ = 45.88, *df* = 8, *p* = .001). In addition to the level of risk and criminogenic needs (*HR* = 1.98, *p* ≤ .001) and the number of prior incarcerations (*HR* = 1.67, *p* ≤ .001), the contextual factors of parole, housing, and employment were statistically significant. Individuals on parole for the index incarceration (*HR* = 1.36, *p* = .038), with housing difficulties (*HR* = 1.43, *p* ≤ .001), or with employment difficulties (*HR* = 1.27, *p* ≤ .001) had higher hazards of any re-incarcerations than their counterparts.

Model 3, which added interaction terms between the presence of SOH and both contextual and individual factors did not significantly improve model fit (*X*^*2*^ = 17.14, *df* = 11, *p* = .104). The significant covariates in the previous model were still significant, indicating individuals with a high level of risk and criminogenic needs (*HR* = 2.01, *p* ≤ .001), a higher number of prior incarcerations (*HR* = 1.60, *p* ≤ .001; quadratic effect was non significant, *p* = .486), released on parole for the index incarceration (*HR* = 1.35, *p* = .046), with housing difficulties (*HR* = 1.39, *p* ≤ .001), or with employment difficulties (*HR* = 1.27, *p* = .002) had higher hazards of any re-incarceration. While the variable indicating the presence of SOH remained not significant, one interaction term, that is not recommeded for participation in a program, was significantly associated with hazards of any re-incarceration, which revealed that the effect of this contextual factor was at least influenced by the presence of SOH. It appears that individuals who were not recommended for participation in a program in the community had lower hazards of any re-incarceration exclusively for those with SOH (*HR* = 0.33, *p* = .003) given both main effects were not significant. Also, individuals with mental health issues, when compared to those without such issues, had higher hazards of any re-incarceration only in Model 3 (*HR* = 1.27, *p* = .046).

#### Re-incarceration for a New Offense

Similar findings were observed for re-incarceration for a new offense (Table 3). Despite the presence of SOH was not significant, both Model 1 (*X*^*2*^ = 84.78, *df* = 4, *p* ≤ .001) and Model 2 (*X*^*2*^ = 125.86, *df* = 12, *p* ≤ .001) were significantly associated with hazards of re-incarceration for a new offense. In the first two models, the level of risk and criminogenic needs (Model 1: *HR* = 3.97, *p* ≤ .001; Model 2: *HR* = 2.89, *p* ≤ .001) and the number of prior incarcerations (Model 1: *HR* = 2.16, *p* ≤ .001; Model 2: *HR* = 1.98, *p* ≤ .001) were significantly and positively associated with hazards of re-incarceration for a new offense. In Model 2, which added contextual factors, individuals released on parole for the index incarceration (*HR* = 0.24, *p* = .002) had significantly lower hazards of re-incarceration for a new offense compared to persons without probation or parole, while persons with housing difficulties (*HR* = 1.58, *p* ≤ .001) and persons with mental health issues (*HR* = 1.42, *p* = .024) had higher hazards of re-incarceration for a new offense than those without such difficulties and issues.

The addition of the interaction terms in Model 3 did not impact the association with re-incarceration for a new offense with the level of risk and criminogenic needs (*HR* = 2.95, *p* ≤ .001), the number of prior incarceration (*HR* = 1.92, *p* ≤ .001; quadratic effect was non significant, *p* = .826), parole for the index incarceration (*HR* = 0.25, *p* = .003), housing difficulties (*HR* = 1.51, *p* = .002), and mental health issues (*HR* = 1.43, *p* = .030). While the main effects were not significant, persons with SOH who were not recommended for participation in a program in the community had lower hazards of re-incarceration for a new offense than the rest of the sample (*HR* = 0.27, *p* = .011). However, Model 3 again did not significantly improved model fit from the previous model (*X*^*2*^ = 12.28, *df* = 11, *p* = .343).

#### Re-incarceration for a Breach of Conditions

Across all models, the presence of SOH was still not significant (Table 4). Among individual factors, only the level of risk and criminogenic needs was significantly and positively associated with hazards of re-incarceration for a breach of conditions in Model 1 (*HR* = 1.80, *p* ≤ .001), Model 2 (*HR* = 1.57, *p* = .015), and Model 3 (*HR* = 1.60, *p* = .015). The addition of contextual factors in Model 2 improved model fit (*X*^*2*^ = 47.90, *df* = 8, *p* ≤ .001). Two contextual factors were significant and associated with higher hazards of re-incarceration for a breach of conditions: parole (*HR* = 2.50, *p* ≤ .001) and employment difficulties (*HR* = 1.42, *p* ≤ .001).

Model 3 did not significantly improve model fit (*X*^*2*^ = 15.52, *df* = 12, *p* = .214): the addition of the interaction terms related to the presence of SOH yielded essentially the same effect for significant individual and contextual factors as the previous model. Unsurprisingly, with regard to significant contextual factors, persons on parole for the index incarceration had higher hazards of re-incarceration for a breach of conditions (*HR* = 2.45, *p* ≤ .001) than those without probation or parole. Persons with employment difficulties compared to individuals without such difficulties also had higher hazards of such an outcome (*HR* = 1.42, *p* ≤ .001).

Consistent with the observation that Model 2 significantly improved model fit relative to the previous model whereas Model 3 did not across the three re-incarceration outcomes, the BIC indicated that Model 2 provided the best trade-off between fit and parsimony. In fact, Model 2, which included the presence of SOH along with both individual and contextual factors, yielded the lowest BIC value for any re-incarceration (9,896.79), re-incarceration for a new offense (4,747.63), and re-incarceration for a breach of conditions (5,151.84). It also showed very strong differences relative to Model 3 (and Model 1) across re-incarceration outcomes according to conventional guidelines (any re-incarceration = 56.04; re-incarceration for a new offense = 52.91; re-incarceration for a breach of conditions = 53.09). These findings indicate that allowing effects to vary according to the presence of SOH in Model 3 reduced overall parsimony without enhancing explanatory value.

## Discussion

The current study examined whether there were contextual factors conducive to re-incarceration outcomes (i.e., any, new offense, breach of conditions) specific for individuals with SOH who had been sentenced to shorter incarceration terms. Survival analyses first estimated the effect of the presence of SOH assuming homogeneous effects of individual as well as contextual factors for the overall sample, and tested whether these effects differed according to the presence or not of SOH. Despite the negative views of individuals with SOH as posing a pronounced threat to public safety, the most generalizable and parsimonious model identified significant effects of contextual factors – while controlling for individual factors – as being conducive to re-incarceration outcomes for the overall sample of young adults. In other words, the effects of contextual (and individual) factors did not differ between those with and without SOH. This is consistent with the findings that the presence of SOH was not associated with hazards of re-incarceration outcomes, let alone higher hazards, and that no contextual or individual factors conducive to re-incarceration were found to be specific to individuals with SOH. Hence, the findings speak more to the homogeneity between young adults with and without SOH in terms of both individual and contextual factor profiles upon community re-entry and in the effects of such factors in shaping re-incarceration outcomes. Ultimately, this resulted in the re-incarceration of nearly three-quarters of the overall sample, largely for nonviolent crimes or back-door sentencing measures occurring within the first year of the average five-year follow-up period, which suggests that young adults sentenced to shorter terms of incarceration face a number of contextual difficulties in attempting to re-enter the community.

### The Precarious Context Upon Community Re-entry

The context upon community re-entry for young adults sentenced to shorter incarceration terms has been considered precarious because analyses suggested that re-incarceration rates for this group, which are higher than those observed in large representative population studies and meta-analyses (e.g., Durose & Antenangeli, 2021; Fazel & Wolf, 2015), result from interaction between individual and contextual forces (Grattet et al., 2011; Visher & Travis, 2003). Young adults returning to the community appear to be poorly equipped to meet their criminogenic and psychosocial needs, a situation exacerbated by the frequent absence of formal surveillance and support interventions upon community re-entry. While more than eight in 10 young adults in the sample had a high level of risk and criminogenic needs, half of the sample re-entered the community without parole or probation, meaning that they did not receive surveillance and support interventions. This raises questions in terms of risk management as young adults released on parole for the index incarceration were less likely to be re-incarcerated for a new offense than persons released without probation or parole. A recent study of a sample of individuals sentenced to shorter incarceration terms in Canada reported similar findings and suggested that the availability of sustained support interventions and access to community resources (e.g., halfway houses) resulting from being on parole contributed to reducing the risk of new offenses (Arbour & Marchand, 2022). This may explain why re-incarceration outcomes for those on probation were not significantly different than for those without probation or parole. Indeed, while probation, unlike parole, may not include support interventions, the data did not make it possible to examine this distinction. However, the lowered hazards of re-incarceration for a new offense for those on parole were simultaneously associated with increased hazards of re-incarceration for a breach of conditions, suggesting to some extent an emphasis on parole surveillance interventions for individuals on conditional release and, by extension, a widening of the criminal justice system’s net (see Cracknell, 2023). This collateral effect of parole may have negative consequences in terms of desistance, especially given the possible psychological effects of re-incarceration (e.g., Turney et al., 2012), even for a short period, as is often the case in breach of parole conditions in the context of shorter incarceration terms (i.e., re-incarceration until the end of the sentence, without additional time). Net widening was also observed in sentencing as, although not associated with hazards of re-incarceration outcomes, individuals with SOH have more frequent and longer probation legal mandates than those without such a history.

In the study sample, probation and especially parole, was unusual compared to what has been observed in general samples of judicialized persons (Commission québécoise des libérations conditionnelles, 2024; Parole Board of Canada, 2024). This alone might have played a critical role in creating a precarious context upon community re-entry as criminogenic and psychosocial needs such as housing, employment, and mental health, which are associated with higher hazards of re-incarceration outcomes, may have remained unaddressed or under-addressed. The lack of interventions may indicate underlying institutional or even systemic problems beyond the reach of the criminal justice system and its practitioners. Several studies have emphasized the importance of surveillance and support interventions whose delivery are tailored to an individual’s level of risk and criminogenic needs (e.g., Andrews et al., 2004; Mailloux et al., 2003), as well as psychosocial needs. The latter have proven to be particularly difficult to meet and address, for incarcerated individuals both with and without SOH, due to the negative social reaction to their community re-entry (e.g., Lopes et al., 2012; Rydberg, 2018). The unmet needs of young adults that contribute to making the context for community re-entry conducive to re-incarceration are not limited to those theoretically associated to criminal behaviors but also include psychosocial needs, which are arguably more important, given their effect on daily functioning, making re-offending almost “inevitable” in the circumstances (Lievesley et al., 2018, p. 18).

### Young Adults, Sentencing, and Community Risk Management

An important part of the context of community re-entry for young adults sentenced to shorter incarceration terms is the assumption that individuals sentenced to such terms, given the low severity of their offense (history), have low levels of risk, criminogenic needs, and psychosocial needs. They are therefore perceived as requiring little or no formal surveillance and support interventions when returning to the community. In Canada, for instance, individuals given longer sentences are systematically provided access to such interventions upon community re-entry, while those with shorter sentences have access to formal surveillance and support only in relation to sentencing (e.g., probation) or conditional release (e.g., parole). These sentencing and conditional release decisions and practises are not solely based on risk and criminogenic needs principles where provision of interventions is tailored to the level of risk and criminogenic needs (see Cortoni & Lafortune, 2009). Research suggests that parole is significantly more likely for individuals who have lower levels of risk and criminogenic needs (Guy et al., 2015; Vacheret & Cousineau, 2005), while probation is generally based on legal and extralegal factors, particularly offense severity and extensiveness of criminal history (Leiber et al., 2018; Sacks & Ackerman, 2014). Neither of these situations is formally linked to individual needs and difficulties upon community re-entry, suggesting that young adults sentenced to shorter incarceration terms are more likely to experience a precarious context upon community re-entry, especially given their individual profiles. While it is possible for persons to seek support on their own, this ability to demonstrate agency is arguably more complex for individuals with SOH, as access to support-oriented resources may be limited by their criminal history (e.g., Ricciardelli et al., 2023).

This precarious context upon community re-entry is particularly concerning when considered in relation to young adults’ need for preparation for re-entry while incarcerated, the timing of release in their psychosocial development, and their pathway to support. Young adults sentenced to shorter incarceration terms generally have a high level of risk and criminogenic needs, a level not well served in terms of effective institutional interventions to reduce re-offending (Arbour et al., 2022). Despite an increasing number of support programs in shorter term correctional institutions, most are not designed for individuals with a high level of risk and criminogenic needs, especially given (a) involvement is voluntary which is more rare for such risk and need profile, (b) the majority are long duration and automatically exclude persons not meeting the sentence length criteria, and (c) the degree of implementation differs from one institution to another resulting in some programs not being available in some places (Giguère & Payant, 2020; Lafortune & Blanchard, 2010). Also, the literature suggests that young adults transitioning to adult life are in a period that is opportune for major life changes, such as desistance from offending (e.g., Arnett, 2000; Laub et al., 1998; Somerville, 2016), but can also lead to persisting criminal behavior if the repercussions of the conviction are perceived important for the person (Public Safety Canada, 2017). Finally, a recent initiative among individuals with shorter incarceration terms found that the criminal justice system was the gateway to seeking psychosocial support for the vast majority of incarcerated persons (Euvrard & Frechette, 2025). This suggests that providing support would be both appreciated and expected upon community re-entry.

### Limitations

This study has certain limitations. First, the presence of some contextual factors is based on the clinical judgement of criminal justice practitioners. However, this limitation is mitigated by the fact that, in Canada, criminal justice practitioners receive extensive training in using risk assessment instruments to ensure a certain degree of standardization and fairness and most have participated in offending and care-related programs dealing with risk assessment and community management tasks as undergraduates (Guay, 2016; Lafortune & Lusignan, 2004). Second, the information available in the correctional files of those in the study sample did not make it possible to determine the nature of the breached condition that led to re-incarceration. Such information would have made it possible to examine whether the behaviors leading to re-incarceration were indicative of, for example, a progression toward desistance, deterioration, or stabilization. Third, despite recommendations for periodic reassessment of dynamic factors, individuals in the sample had not undergone such reassessment. As a result, any change in community intervention needs and psychosocial needs was not detected, meaning the study design was forced to assume they were relatively stable.

## Conclusion

This study examined the context of community re-entry for young adults with SOH given sentences of under two years to determine if there are aspects of the experience that are conducive to re-incarceration. The findings suggest that their experience with the context upon community re-entry is similar to that of other young adults, providing foundations for bridging literature specific to sexual offending and that of level of risk and criminogenic needs, social reintegration and risk of re-offense in general samples of judicialized persons in jurisdictions favoring community risk management. This contribution highlights the high prevalence and quickness of re-incarceration among young adults with and without SOH as a result of a precarious context upon community re-entry, exacerbated by their high levels of risk and criminogenic needs, which are frequently not addressed through formal surveillance and support interventions for those serving shorter incarceration sentences. These findings must not, however, be taken as evidence that decisions and practices concerning community re-entry should be blindly based on level of risk and criminogenic needs. Instead, to prevent re-incarceration, they show the importance of tailoring surveillance and support interventions to simultaneously address individual levels of risk and criminogenic needs as well as contextual factors, which might be overlooked in community re-entry interventions based on risk and criminogenic needs principles. Indeed, certain basic needs, such as psychosocial needs, can influence community re-entry in a different way than criminogenic needs. While the latter provide information about the factors contributing to criminal behavior and re-offending, psychosocial needs perhaps provide information about readiness for community re-entry, and by extension, vulnerability to criminogenic needs, behavioral patterns prior to incarceration, and known pathways to re-offending.

More research is needed to unravel the scope, nature, and impact on re-offense of criminogenic needs, psychosocial needs, and the effect of surveillance as well as support interventions specific to persons with SOH. In fact, the combination of surveillance and support interventions for individuals in the sample did not make it possible to determine whether those with and without SOH encountered similar precarious re-entry contexts in terms of community correction, psychosocial needs, social reaction, and re-offending, or whether support interventions tailored by criminal justice practitioners to persons with SOH or even surveillance interventions specific to this group (i.e., non-public registry and prohibition orders) affected re-incarceration in one direction or another. While these interventions specific to individuals with SOH exist, their effect on re-offending and social reintegration upon community re-entry is unknown in Canada.

## Notes

1. The term ‘shorter’ is used throughout in comparison with longer sentences, particularly federal, which in Canada are for a minimum of two years and can go up to several decades. The authors are in no way trying to minimize the impact of incarceration terms of less than two years.

2. Employment difficulties, although an item of the actuarial component of the LS/CMI, is part of a list of statements related to generalized troubles (e.g., “Financial problems”, “Nonrewarding”), which, if sufficiently numerous, can lead to an increase of one point in the actuarial score. However, several individuals who noted employment difficulties did not see their actuarial score change. The opposite is also true, that individuals without employment difficulties saw their score increase by one point on this item, which makes its use suitable and not necessarily redundant.
